# A rare case of Langerhans cell histiocytosis with unexplained endocrine dysfunction and extensive skeletal involvement in a Pediatric patient

**DOI:** 10.1093/omcr/omaf155

**Published:** 2025-09-15

**Authors:** Carlos Díaz Q, Marcos Orellana, Rolando Chajon, José Valenzuela, Pedro Chajon

**Affiliations:** Department of Research, Universidad Francisco Marroquín, 13 Av, Guatemala City 01011; Department of Research, Universidad Francisco Marroquín, 13 Av, Guatemala City 01011; Department of Research, Universidad Francisco Marroquín, 13 Av, Guatemala City 01011; Department of Research, Universidad Francisco Marroquín, 13 Av, Guatemala City 01011; Department of Research, Universidad Francisco Marroquín, 13 Av, Guatemala City 01011

**Keywords:** rheumatology, paediatrics

## Abstract

Langerhans cell histiocytosis (LCH) is a rare disorder in which Langerhans cells infiltrate various organs, causing damage to tissues. This case describes a 6-year-old male diagnosed with LCH, who presented with worsening symptoms of fatigue, excessive thirst, polyuria, and unexplained weight gain. Radiographic findings revealed extensive osteolytic lesions in the skull and long bones, consistent with LCH. Additionally, the patient developed significant endocrine dysfunction, including hypothyroidism, diabetes insipidus, and adrenal insufficiency, as confirmed through hormonal assays and imaging. The case emphasizes the importance of considering endocrine dysfunction in patients with LCH and underscores the role of a multidisciplinary approach in managing complex cases. Hormonal replacement therapy, along with chemotherapy for LCH, was initiated, and the patient showed clinical improvement.

## Introduction

Langerhans cell histiocytosis (LCH) is a rare disorder characterized by the proliferation of Langerhans cells, a type of dendritic cell that infiltrates various tissues such as bones, skin, liver, spleen, and lungs. The incidence of LCH is estimated to be approximately 5 cases per million children per year, though the disorder can also affect adults, though it is less common in this population [1]. The clinical manifestations of LCH vary depending on the organs involved, with bone lesions being the most frequent feature. However, in rare cases, LCH can also cause significant endocrine dysfunction, including involvement of the hypothalamic–pituitary–adrenal (HPA) axis [2]. This case report presents a 6-year-old male patient with LCH who developed extensive osteolytic lesions along with unexplained endocrine dysfunction, including hypothyroidism, diabetes insipidus, and adrenal insufficiency. The case highlights the importance of early detection and multidisciplinary management for patients with LCH and multiple system involvement.

## Case presentation

**Figure 1:**
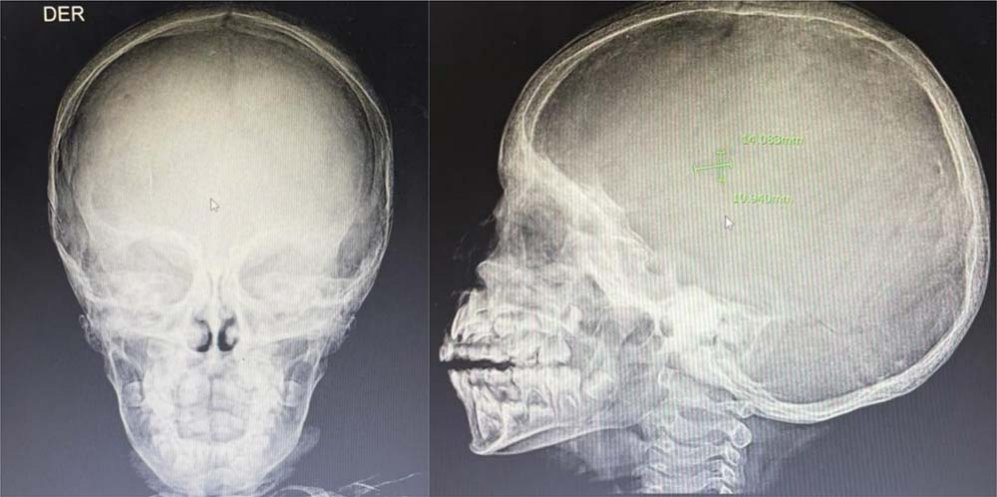
Skull radiograph showing Calvarial involvement in LCH. Lateral radiograph of the skull showing multiple osteolytic lesions scattered across the calvarium, with the largest measuring 1.4 × 1.0 cm in the maxillary region. The lesions exhibit irregular but well-defined margins, a narrow zone of transition, and a geographic pattern of bone loss, consistent with Langerhans cell histiocytosis. Evidence of diffuse cranial hyperostosis is also noted.

**Figure 2:**
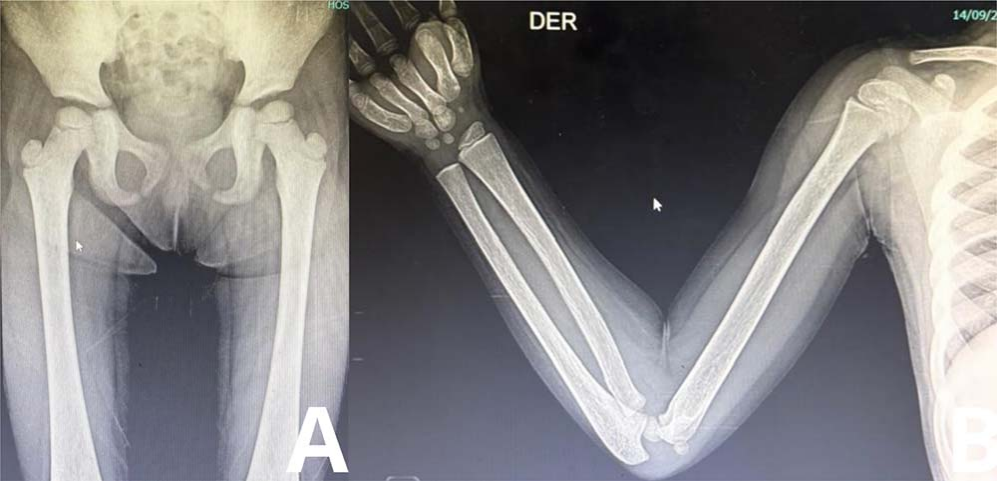
Radiographic evaluation of axial and appendicular skeleton (A-B). A. Anteroposterior radiograph of the lower abdomen and thighs showing multiple well-defined osteolytic lesions affecting the femurs bilaterally, with a geographic pattern and narrow transition zones. Mild cortical thinning is observed, particularly in the distal femoral metaphyses, suggestive of chronic LCH involvement. B. Anteroposterior radiograph of the right arm demonstrating a lytic lesion in the mid-diaphysis of the humerus with preserved cortical contour and no periosteal reaction, consistent with a non-aggressive process typical of LCH.

**Figure 3:**
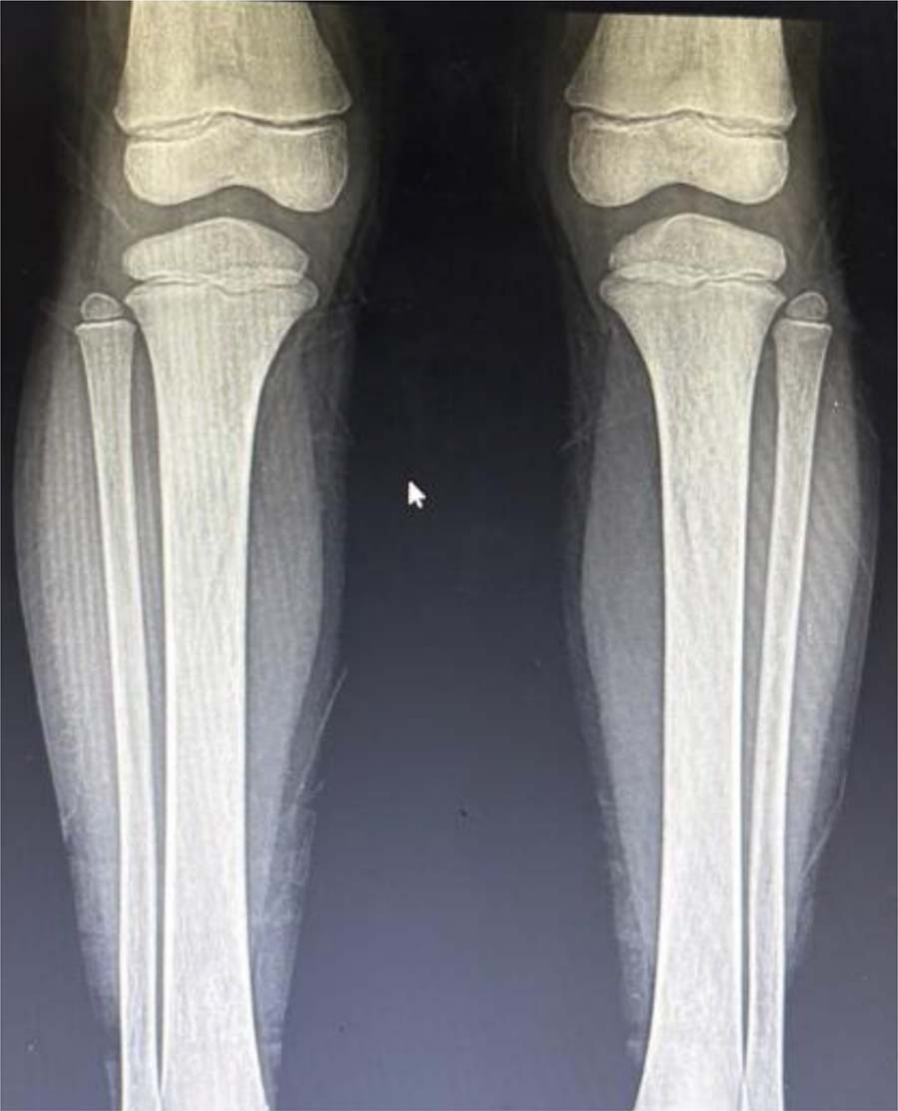
Lower extremity long bone involvement in LCH. Anteroposterior radiograph of both lower extremities revealing multiple bilateral osteolytic lesions in the femoral shafts and metaphyses. The lesions display a geographic pattern with well-defined, irregular borders and a narrow zone of transition. Cortical integrity is preserved, and no periosteal reaction is observed. These findings are consistent with chronic skeletal involvement in Langerhans cell histiocytosis.

A 6-year-old male with a prior diagnosis of Langerhans cell histiocytosis (LCH) presented for a follow-up evaluation with worsening symptoms over the past several months. He had initially been diagnosed with LCH at the age of 3, following evaluation for intermittent bone pain, particularly in the maxillary region. Radiographic studies, including X-rays and CT scans, revealed multiple osteolytic lesions in the calvarium, temporal bone, and long bones specifically in the femur and humerus with features consistent with LCH. (Figs 1–3).

The osteolytic lesions in the long bones exhibited a geographic pattern with narrow transition zones and signs of endosteal scalloping. These lesions contributed to significant bone fragility, evidenced by a history of two atraumatic fractures during routine activities. The largest lesion in the maxilla measured 1.4 x 1.0 cm, with irregular borders and a narrow zone of transition, consistent with active bone destruction. Recent radiographs showed a marked decrease in the size of several cranial lesions, suggesting a partial response to previous chemotherapy.

Despite ongoing systemic treatment, the patient’s condition deteriorated over the preceding six months. His parents reported progressive fatigue, excessive thirst, frequent urination, and unexplained weight gain, despite no changes in diet. On physical examination, the child appeared pale, exhibited dry skin, and had signs of cold intolerance. Notably, his blood pressure was elevated for his age, and delayed deep tendon reflexes were present. A subtle facial asymmetry due to a protruding maxillary lesion was observed.

Laboratory tests showed elevated thyroid-stimulating hormone (TSH) and decreased free thyroxine (T4), indicating hypothyroidism. A water deprivation test confirmed the diagnosis of diabetes insipidus, and serum cortisol levels were significantly reduced, suggesting adrenal insufficiency. MRI of the brain demonstrated slight enlargement of the pituitary gland, while abdominal CT revealed adrenal atrophy. These findings pointed toward hypothalamic–pituitary–adrenal axis involvement by LCH, leading to multisystem endocrine dysfunction.

The patient was started on hormone replacement therapy, including levothyroxine for hypothyroidism, hydrocortisone for adrenal insufficiency, and desmopressin for central diabetes insipidus. This multidisciplinary approach helped stabilize his endocrine symptoms and improved his overall quality of life while ongoing monitoring of bone involvement continued through imaging.

The patient was born to non-consanguineous parents; however, there was a notable family history of autoimmune thyroid disease and early-onset diabetes in maternal relatives, raising suspicion of a possible genetic predisposition to endocrine dysfunction.

Although access to advanced molecular diagnostics was limited, targeted genetic analysis confirmed the presence of the BRAF V600E mutation, a variant commonly associated with more aggressive forms of LCH.

## Discussion

Langerhans cell histiocytosis is a rare, multi-system disorder that primarily affects children, though it can also occur in adults. The disease is characterized by the abnormal proliferation of Langerhans cells, which can infiltrate a variety of organs, leading to both localized and systemic symptoms. While bone involvement is most common, LCH can also cause significant endocrine dysfunction, particularly when the hypothalamic–pituitary–adrenal axis is involved. The patient’s presentation of fatigue, polyuria, and weight gain, alongside radiographic findings of osteolytic lesions, raised suspicion for an underlying endocrine disorder [3].

The pathophysiology of endocrine dysfunction in LCH is thought to be related to the infiltration of Langerhans cells into the hypothalamus, pituitary gland, and adrenal glands. This can lead to a variety of hormonal imbalances, including diabetes insipidus (due to posterior pituitary involvement), hypothyroidism (due to pituitary or thyroid involvement), and adrenal insufficiency (due to adrenal infiltration). This patient’s case illustrates the need for a high degree of clinical suspicion when LCH presents with multisystem involvement, particularly when endocrine symptoms emerge.

Skeletal involvement is the most common manifestation of LCH, occurring in up to 80% of pediatric cases, particularly affecting the skull and long bones. Endocrine dysfunction, though less common, is reported in approximately 20%–25% of patients, with diabetes insipidus being the most frequent due to posterior pituitary involvement. Although skin lesions are commonly seen in LCH, up to 40% of patients—especially those with multisystem disease—may present without cutaneous involvement, making diagnosis more challenging and often delaying recognition of systemic features [4].

In addition to the typical skeletal involvement, endocrine dysfunction in LCH requires a comprehensive diagnostic approach. Hormonal assays, including TSH, cortisol, and antidiuretic hormone (ADH) levels, along with imaging studies such as MRI and CT scans, are crucial for confirming the diagnosis [5 , 6]. The water deprivation test is an essential tool for diagnosing diabetes insipidus, as it helps differentiate it from other causes of polyuria.

Treatment of LCH involves a combination of chemotherapy and supportive care. Common chemotherapeutic agents used in LCH include vinblastine and prednisone, which help to reduce the proliferation of Langerhans cells and alleviate symptoms. Hormonal replacement therapy is essential in managing the endocrine dysfunction, and the patient was initiated on levothyroxine for hypothyroidism, hydrocortisone for adrenal insufficiency, and desmopressin for diabetes insipidus [7].

The treatment for this pediatric patient with Langerhans Cell Histiocytosis (LCH) involved systemic corticosteroids, chemotherapy with vinblastine and prednisolone, and bisphosphonates to manage osteolytic lesions. Endocrine dysfunction was addressed with thyroid and growth hormone replacements [8]. Targeted therapies, such as vemurafenib, were also considered for cases with the BRAF V600E mutation. The approach was tailored to the patient’s needs and coordinated by a multidisciplinary team, ensuring careful monitoring and adjustment based on disease progression and organ involvement [9].

Multidisciplinary care is essential in managing complex LCH cases with multisystem involvement. Endocrinologists, oncologists, and radiologists must work closely together to monitor the patient’s disease progression and ensure appropriate management of both the underlying LCH and its complications. Endocrine manifestations in LCH often require long-term follow-up, as deficiencies can persist or progress. Regular hormonal evaluations and appropriate hormone replacement therapy adjusted as the child grows—are key to effective management. Given the rarity of endocrine dysfunction in LCH, ongoing research into the pathophysiology and optimal treatment strategies is warranted.

## Conclusion

This case highlights the complex nature of Langerhans cell histiocytosis and its potential to cause both extensive skeletal involvement and significant endocrine dysfunction. The patient’s development of hypothyroidism, diabetes insipidus, and adrenal insufficiency underscores the need for early recognition of endocrine disturbances in patients with LCH. Hormonal replacement therapy, alongside chemotherapy for LCH, can help manage these complications and improve the patient’s quality of life. This case serves as a valuable reminder of the importance of a multidisciplinary approach in the management of rare diseases like LCH, where both systemic involvement and endocrine dysfunction can complicate the clinical picture. Continued monitoring and individualized care are crucial to optimizing outcomes for these patients.

## Data Availability

All data supporting this case report are included in the article, with no additional data available due to patient confidentiality.
